# Editorial: Al-Induced and -Activated Signals in Aluminium Resistance

**DOI:** 10.3389/fpls.2022.925541

**Published:** 2022-06-22

**Authors:** Hiroyuki Koyama, Chao-Feng Huang, Miguel A. Piñeros, Yoko Yamamoto

**Affiliations:** ^1^Applied Biological Sciences, Gifu University, Gifu, Japan; ^2^Shanghai Center for Plant Stress Biology, Center for Excellence in Molecular Plant Sciences, Chinese Academy of Sciences (CAS), Shanghai, China; ^3^Robert W. Holley Center for Agriculture & Health, USDA Agricultural Research Service (USDA-ARS), Ithaca, NY, United States; ^4^Institute of Plant Science and Resources, Okayama University, Kurashiki, Japan

**Keywords:** aluminum, signaling, STOP1, Arabidopsis, legume

As phytotoxic aluminum (Al) appears in the acid soil at pH <5.5 (Kobayashi et al., 2013), plants have evolved resistance mechanisms to cope with the Al rhizotoxicity (Daspute et al., 2017). Research in this field has identified various constitutive and induceble Al resistance mechanisms that protect Al-sensitive cells in the roots (Daspute et al., 2017). For example, exudation of organic acid (OA) from the roots protects the sensitive cells by chelating the phytotoxic Al^3+^ to less toxic Al-OA chelated compounds. This OA exudation is controlled by both Al-activated and Al-inducible mechanisms, as reported for the malate transporter ALMT1 (ALUMINUM ACTIVATED MALATE TRANSPORTER 1) in various plant species (Wu et al., 2018). In addition, recent studies identified pleiotropic roles of Al tolerant proteins such as SENSITIVE TO PROTON RHIZOTIXICITY1 (STOP1) (Koyama et al., 2021; Sadhukhan et al., 2021b) and ALMT1 (Wu et al., 2018). The level of such proteins play crucial role to manage Al tolerance and growth response (Fang et al., 2021a). This Research Topic, “*Al-Induced and -Activated Signals in Aluminium Resistance*,” aimed to enrich our knowledge, enabling a better understanding of the complexity underlining Al resistance mechanisms. The Research Topic contains six original research articles, including three studies using the model species Arabidopsis and three others in crops species, namely tomato, lentil, and cicer.

The studies in Arabidopsis focused on the transcription factor STOP1-regulated system. This extensively studied zinc finger transcription factor regulates the expression of several genes, such as *AtALMT1*, critical for Al resistance (Sadhukhan et al., 2021b). *AtALMT1*-expression requires direct binding of STOP1 to the promoter, while the activity and amounts of STOP1 are regulated by complex mechanisms, including posttranslational modification. Huang's group reported several signaling proteins involved in the process of controlling STOP1 using *rae* (REGULATION OF ATALMT1 EXPRESSION) mutants with altered *AtALMT1* expression levels. For example, RAE1, an F-box protein component of a SCF-type E3 ligase complex, alters *AtALMT1* expression by degradation of STOP1 through ubiquitination (Zhang et al., 2019), while the SUMO protease RAE5/EARLY IN SHORT DAYS 4 (ESD4) and SUMO E3 ligase SIZ1 can influence *AtALMT1* expression by deSMOylation and SUMOylation of STOP1, respectively (Fang et al., 2021b; Xu et al., 2021). In addition, RAE3, a core component of the THO/TREX complex, regulates *AtALMT1* expression by modulating nucleocytoplasmic *STOP1* mRNA export and consequently influencing the STOP1 protein accumulation. In the current Topic, Zhu et al. provided additional information on RAE2/TEX1, another core component of the THO complex, which is also involved in the regulation of Al resistance and low Pi response by regulating *AtALMT1* expression level. Interestingly, *rae2* did not show defective nucleocytoplasmic *STOP1* mRNA export but accumulated less STOP1 protein and consequently had reduced expression of *AtALMT1*. It appears that THO-complex, possibly conjugated with mRNA, plays essential role in regulating STOP1's downstream Al resistant genes and development.

On the other hand, Agrahari et al. implemented a Genome-Wide Association (GWAS) to explore the upstream region of STOP1-regulated Al resistant genes. POLYGARACTRONASE INHIBITOR PROTEIN 1 (PGIP1), a stabilizing cell wall pectin, possesses STOP1 binding cis-elements in its promoter and showed Al-inducible expression. eGWAS identified a STOP1-independent NO signaling pathway and a STOP1-dependent regulation in the phosphatidylinositol (PI) signaling pathway, involved in the regulation of *PGIP1* expression under Al stress. The former included one of the TRX superfamily proteins, while the latter included NAC027 and an R-R-type MYB transcription factors. A similar eGWAS approach provided further genetic evidence that PI signaling is also involved in the regulation of the Al-resistance gene *ALUMINUM SENSITIVE3* (*ALS3*), possibly through the activation of STOP1 (Sadhukhan et al., 2021a). Furthermore, that study identified the involvement of a Ca signaling pathway in the Al inducible *ALS3* expression, which would be independent of the STOP1 pathway.

Desnos and coworkers previously studied the activation of STOP1 in response to P-deficiency in gel medium, providing evidence that the STOP1-activation could occur in the absence of Al stress (Balzergue et al., 2017). In the current topics, the authors (Le Poder et al.) discovered that the STOP1-AtALMT1 pathway could be activated by Al at a level that does not inhibit root growth of the Al-hypersensitive mutant *stop1*, suggesting that Al signaling can be uncoupled from its toxicity. Furthermore, they found that Al and iron (Fe) can play a synergistic role in activating the STOP1-AtALMT1 pathway. These three studies in Arabidopsis provided new insight into the STOP1-dependent Al signaling, including a very sensitive response to Al, a coordinated regulation with other signaling pathways (i.e., PI, NO, and Ca signaling), and the complex regulation at the STOP1 level *via* the THO-complex. On the other hand, the regulation of various stress tolerance responses (e.g., Al, proton, P-deficiency hypoxia, and drought) by STOP1 could be referred to as pleiotropy of stress tolerance reported in Arabidopsis and other plants.

Three other manuscripts describe Al signaling and tolerance responses in crop plants. The AAEs (acyl-activating enzymes superfamily) are involved in multiple metabolic pathways, with some of them identified as being related to Al tolerance, Jin et al. studied the expression of AAEs in tomato under aluminum stress. A synteny analysis of AAEs in tomato and Arabidopsis and cis-element predictions suggested that some Al-inducible *AAE*s carry STOP1-binding motifs in the promoter. Lentil is one of India's oldest cultivating legume and is currently grown as a winter legume. Singh et al. studied inter-and intraspecies transcriptomes that identify different metabolic pathways regulating Al tolerance in lentil. The main upregulated and differentially expressed genes (DEGs) identified under stress conditions are underline organic acid synthesis and exudation, production of antioxidants, callose synthesis, protein degradation, and phytohormone- and calcium-mediated signaling pathways. These genes and pathways are homologous to those Al-inducible processes described in model plants. The study also identified several transcription factors upregulated in tolerant varieties and wild species, probably associated with auxin signal. These findings represent a novel and useful target to improve lentils' Al tolerance using expression-level polymorphisms. Finally, Vance et al. reported Al resistance in wild cicer, a relative species of chickpea. The authors conducted a large-scale screening of Al and low pH tolerance using relative root elongation as a tolerance index, resulting in the identification of resistant cultivars associated with the original locations. Although this study did not focus on Al signaling, it provides novel information on Al resistant varieties of chickpea, a commercially important legume.

This Research Topic shine shed some light in compiling crucial information for a better understanding of the complex signaling involved in the response to Al stress. We welcome readers to explore the six research papers for deeper analysis of their findings.

## Author Contributions

All authors listed have made a substantial, direct, and intellectual contribution to the work and approved it for publication.

## Funding

HK was funded by JSPS (KAKENHI and 21H02088).

## Conflict of Interest

The authors declare that the research was conducted in the absence of any commercial or financial relationships that could be construed as a potential conflict of interest.

## Publisher's Note

All claims expressed in this article are solely those of the authors and do not necessarily represent those of their affiliated organizations, or those of the publisher, the editors and the reviewers. Any product that may be evaluated in this article, or claim that may be made by its manufacturer, is not guaranteed or endorsed by the publisher.
